# Supplementary material for: Pyruvate Dehydrogenase Kinase 1 inhibition mediated oxidative phosphorylation enhancement in cartilage promotes osteoarthritis progression
Source: BMC Musculoskelet Disord. 2023 Jul 20;24:597. doi: 10.1186/s12891-023-06585-6 (PMC10357736; doi:10.1186/s12891-023-06585-6)
Supplement: Supplementary file 1 — Additional file 1. [file 12891_2023_6585_MOESM1_ESM.zip › BP.Gsea.1653623667859/GOBP_REGULATION_OF_SUPEROXIDE_METABOLIC_PROCESS.html]

Details for gene set GOBP\_REGULATION\_OF\_SUPEROXIDE\_METABOLIC\_PROCESS[GSEA]

|  || Dataset | 55457\_collapsed\_to\_symbols.cls.cls #OA\_versus\_normal.cls.cls #OA\_versus\_normal\_repos |
| Phenotype | cls.cls#OA\_versus\_normal\_repos |
| Upregulated in class | OA |
| GeneSet | GOBP\_REGULATION\_OF\_SUPEROXIDE\_METABOLIC\_PROCESS |
| Enrichment Score (ES) | 0.42111912 |
| Normalized Enrichment Score (NES) | 1.5117759 |
| Nominal p-value | 0.035714287 |
| FDR q-value | 0.3602993 |
| FWER p-Value | 1.0 |
Table: GSEA Results Summary

  

Fig 1: Enrichment plot: GOBP\_REGULATION\_OF\_SUPEROXIDE\_METABOLIC\_PROCESS      
 Profile of the Running ES Score & Positions of GeneSet Members on the Rank Ordered List

  

| SYMBOL | TITLE | RANK IN GENE LIST | RANK METRIC SCORE | RUNNING ES | CORE ENRICHMENT || 1 | FBLN5 | fibulin 5 [Source:HGNC Symbol;Acc:HGNC:3602] | 69 | 0.958 | 0.1217 | Yes |
| 2 | CYBA | cytochrome b-245 alpha chain [Source:HGNC Symbol;Acc:HGNC:2577] | 501 | 0.658 | 0.1754 | Yes |
| 3 | ITGB2 | integrin subunit beta 2 [Source:HGNC Symbol;Acc:HGNC:6155] | 827 | 0.562 | 0.2246 | Yes |
| 4 | GSTP1 | glutathione S-transferase pi 1 [Source:HGNC Symbol;Acc:HGNC:4638] | 1260 | 0.465 | 0.2527 | Yes |
| 5 | NFE2L2 | "nuclear factor, erythroid 2 like 2 [Source:HGNC Symbol;Acc:HGNC:7782]" | 2034 | 0.357 | 0.2401 | Yes |
| 6 | TYROBP | transmembrane immune signaling adaptor TYROBP [Source:HGNC Symbol;Acc:HGNC:12449] | 2058 | 0.355 | 0.2854 | Yes |
| 7 | PRKCD | protein kinase C delta [Source:HGNC Symbol;Acc:HGNC:9399] | 2237 | 0.335 | 0.3159 | Yes |
| 8 | BST1 | bone marrow stromal cell antigen 1 [Source:HGNC Symbol;Acc:HGNC:1118] | 2319 | 0.324 | 0.3525 | Yes |
| 9 | SOD1 | superoxide dismutase 1 [Source:HGNC Symbol;Acc:HGNC:11179] | 2413 | 0.313 | 0.3868 | Yes |
| 10 | TGFB1 | transforming growth factor beta 1 [Source:HGNC Symbol;Acc:HGNC:11766] | 2490 | 0.303 | 0.4211 | Yes |
| 11 | GNAI3 | G protein subunit alpha i3 [Source:HGNC Symbol;Acc:HGNC:4387] | 3694 | 0.186 | 0.3523 | No |
| 12 | MAPT | microtubule associated protein tau [Source:HGNC Symbol;Acc:HGNC:6893] | 3976 | 0.158 | 0.3514 | No |
| 13 | SYK | spleen associated tyrosine kinase [Source:HGNC Symbol;Acc:HGNC:11491] | 4084 | 0.147 | 0.3626 | No |
| 14 | AGT | angiotensinogen [Source:HGNC Symbol;Acc:HGNC:333] | 4407 | 0.120 | 0.3536 | No |
| 15 | ACP5 | "acid phosphatase 5, tartrate resistant [Source:HGNC Symbol;Acc:HGNC:124]" | 4456 | 0.117 | 0.3653 | No |
| 16 | CRP | C-reactive protein [Source:HGNC Symbol;Acc:HGNC:2367] | 5154 | 0.059 | 0.3190 | No |
| 17 | PON3 | paraoxonase 3 [Source:HGNC Symbol;Acc:HGNC:9206] | 5340 | 0.045 | 0.3106 | No |
| 18 | DHFR | dihydrofolate reductase [Source:HGNC Symbol;Acc:HGNC:2861] | 5805 | 0.008 | 0.2756 | No |
| 19 | ITGAM | integrin subunit alpha M [Source:HGNC Symbol;Acc:HGNC:6149] | 5820 | 0.007 | 0.2755 | No |
| 20 | CLEC7A | C-type lectin domain containing 7A [Source:HGNC Symbol;Acc:HGNC:14558] | 7090 | -0.087 | 0.1884 | No |
| 21 | CD36 | CD36 molecule [Source:HGNC Symbol;Acc:HGNC:1663] | 7985 | -0.154 | 0.1394 | No |
| 22 | F2RL1 | F2R like trypsin receptor 1 [Source:HGNC Symbol;Acc:HGNC:3538] | 8631 | -0.203 | 0.1161 | No |
| 23 | GNAI2 | G protein subunit alpha i2 [Source:HGNC Symbol;Acc:HGNC:4385] | 8686 | -0.208 | 0.1395 | No |
| 24 | AATF | apoptosis antagonizing transcription factor [Source:HGNC Symbol;Acc:HGNC:19235] | 8935 | -0.227 | 0.1503 | No |
| 25 | GCH1 | GTP cyclohydrolase 1 [Source:HGNC Symbol;Acc:HGNC:4193] | 9465 | -0.271 | 0.1451 | No |
| 26 | BMP7 | bone morphogenetic protein 7 [Source:HGNC Symbol;Acc:HGNC:1074] | 9544 | -0.278 | 0.1758 | No |
| 27 | FPR2 | formyl peptide receptor 2 [Source:HGNC Symbol;Acc:HGNC:3827] | 9857 | -0.307 | 0.1923 | No |
| 28 | CD177 | CD177 molecule [Source:HGNC Symbol;Acc:HGNC:30072] | 10114 | -0.332 | 0.2164 | No |
Table: GSEA details [plain text format]

  

Fig 2: GOBP\_REGULATION\_OF\_SUPEROXIDE\_METABOLIC\_PROCESS      
 Blue-Pink O' Gram in the Space of the Analyzed GeneSet

  

Fig 3: GOBP\_REGULATION\_OF\_SUPEROXIDE\_METABOLIC\_PROCESS: Random ES distribution      
 Gene set null distribution of ES for **GOBP\_REGULATION\_OF\_SUPEROXIDE\_METABOLIC\_PROCESS**
